# Anesthetic Personnel's Experiences and Perspectives With a National Standard for Safeguarding Anesthesia Practice: A Cross-Sectional Study in Norway

**DOI:** 10.1155/anrp/6302974

**Published:** 2025-07-17

**Authors:** Ann-Chatrin Linqvist Leonardsen, Arvid Steinar Haugen, Johan Ræder, Therese Jenssen Finjarn, Erik Isern, Elin Kismul Aakre, Anne Marie Gran Bruun, Kristoffer Hennum, Jan Petter Ramstad, Tina Sand, Cathrine Saltnes, Svein Arne Monsen

**Affiliations:** ^1^Department of Health, Care and Organisation, Ostfold University College, Halden, Norway; ^2^Department of Health and Social Sciences, University of Southeastern Norway, Kongsberg, Norway; ^3^Department of Anesthesia, Ostfold Hospital Trust, Kalnes, Østfold, Norway; ^4^Department of Anesthesia and Intensive Care, Haukeland University Hospital, Bergen, Norway; ^5^Institute of Health Sciences, Acute and Critical Care, Oslo Metropolitan University, Oslo, Norway; ^6^Institute of Clinical Medicine, University of Oslo, Oslo, Norway; ^7^Department of Anesthesia, Bærum Hospital, Bærum, Norway; ^8^Department of Anesthesiology, St.Olavs' Hospital, Trondheim, Norway; ^9^Department of Anesthesiology, Ringerike Hospital, Hønefoss, Norway; ^10^Department of Anesthesia, Gjøvik Hospital, Gjøvik, Norway; ^11^Department of Anesthesiology, Nord University Hospital, Tromsø, Norway; ^12^Department of Anesthesiology, Nordland Hospital, Bodø, Norway

## Abstract

**Background:** Globally, anesthesiologists and nurse anesthetists collaborate closely during anesthesia administration. However, there is a scarcity of guidelines detailing the division of tasks and responsibilities between these two professions. The Norwegian Standard for the Safe Practice of Anesthesia (NSA) was developed jointly by the Norwegian Association of Anesthesiologists and the Norwegian Association of Nurse Anesthetists as a consensus guideline to safeguard satisfactory anesthetic practice. This study aimed to explore the experiences and perspectives of anesthesiologists and nurse anesthetists regarding the NSA.

**Methods:** The study employed a cross-sectional, observational design, utilizing a questionnaire. A purposive sampling strategy was employed, inviting all members of the two associations (*N* = 3300) to participate in a web-based survey. Data were analyzed using the Statistical Package for the Social Sciences, Version 28. Descriptive statistics and independent samples *t*-tests were utilized to analyze the data. A two-sided *p* value of ≤ 0.05 was considered statistically significant.

**Results:** In total, 823 respondents (24.9%) completed the questionnaire in September 2024. The results indicate several areas for improvement to achieve the recommended standards of anesthetic practice as outlined by the NSA. Statistically significant differences were observed between responses from anesthesiologists and nurse anesthetists. Approximately two-thirds of respondents reported the NSA as relevant to their daily work, and between 13 and 30 percent had experienced or were aware of situations where the standard was utilized in root cause analyses of adverse anesthetic events.

**Conclusion:** The findings suggest that the NSA is employed in clinical practice. However, assuming the NSA ensures satisfactory anesthetic practice, there are several areas requiring improvement. Given the overlapping roles and responsibilities of anesthesiologists and nurse anesthetists, the NSA may serve as a model for similar guidelines in other countries.

## 1. Background

Globally, anesthesia care delivery models demonstrate considerable variation. These models include anesthesiologists working independently, anesthesiologists supervising nonphysician anesthesia personnel, nonphysician anesthesia personnel operating autonomously, and scenarios where surgical personnel concurrently perform surgeries and administer anesthesia [1]. Such diversity is also present within regions of individual countries. For instance, in the United States, although 14 states require some degree of physician oversight, three states allow nurse anesthetists to practice entirely independently [1]. In areas such as the United States, Asia, and Europe, nurse anesthetists commonly collaborate closely with anesthesiologists during anesthesia administration [2].

Despite the establishment of clinical standards and guidelines by esteemed organizations such as the American Society of Anesthesiologists (ASA) [3], the Royal College of Anesthetists [4], the World Health Organization–World Federation of Societies of Anesthesiologists (WHO-WFSA) [5], and the International Federation of Nurse Anesthetists (IFNA) [6], there is a lack of comprehensive international consensus regarding collaboration models between anesthetists and nurse anesthetists. These guidelines do not provide detailed protocols on how these professionals should collaborate, nor do they outline the distribution of tasks and responsibilities.

In Norway, the Norwegian Association of Anesthesiologists (NAA) and the Norwegian Association of Nurse Anesthetists (NANA) addressed this need by developing a standard for safe anesthesia practices, known as the Norwegian Standard for the Safe Practice of Anesthesia (NSA), in 1991. This standard was created to safeguard anesthetic practices and define the division of tasks and responsibilities between anesthetists and nurse anesthetists. The NSA is periodically updated to align with current legislation, advancements in medical and technological practices, and evolving clinical standards, with the most recent revision taking place in 2024 [7]. It serves as normative guidelines applicable to all anesthesia personnel, irrespective of geographical and organizational contexts, ensuring patient safety and maintaining satisfactory anesthetic practices in Norway.

Currently, there is no similar common standard or guideline internationally concerning patient safety or task-sharing between anesthetists and nurse anesthetists. This highlights a significant knowledge gap in the field of anesthesia care models. The purpose of this study was to further explore this gap and assess the efficacy and impact of the NSA in the Norwegian context, with the intention of contributing to the development of standardized global practices. As such, the aim was to explore the experiences and perspectives of anesthesiologists and nurse anesthetists regarding the NSA.

## 2. Methods

### 2.1. Design

The study employed a descriptive, cross-sectional design using a questionnaire. This approach allowed researchers to collect data at a single point in time, offering a snapshot of the current situation or phenomena. Such a design is particularly useful when seeking to understand existing conditions, opinions, or behaviors within a specific population, as was the case in examining nurse anesthetists' and anesthesiologists' experiences and perspectives with the NSA. The study adheres to the Strengthening the Reporting of Observational Studies in Epidemiology (STROBE) statement guidelines [8].

In Norway, a nurse anesthetist is a registered nurse with a master's degree or postgraduate education as a nurse anesthetist.

### 2.2. Participants

A purposive sampling strategy was implemented. All members of the NAA (*n* = 1500) and NANA (*n* = 1800) were invited to participate in the study. Although there is no national count of registered consultant anesthetists or nurse anesthetists, our experience suggests that most are members of the NAA or NANA, respectively. Inclusion criteria encompassed individuals who were nurse anesthetists or anesthesiologists. There were no exclusion criteria. A purposive recruitment strategy was utilized, with all members of the two associations invited via email, followed by one reminder, during the period from August 31 to September 30, 2024. No method was employed for calculating the appropriate sample size.

### 2.3. Data Collection

The development of the questionnaire for this study was rigorously undertaken to ensure its validity. Initially crafted by the first author, a nurse anesthetist with extensive academic credentials, including a PhD, the questionnaire was anchored in the NSA's chapters and insights from the 2024 revision process. This foundation aimed to create a tool that accurately reflects the NSA's scope and objectives.

To enhance its validity, the questionnaire underwent a comprehensive review by a diverse working group involved in the 2024 NSA revision. This group comprised six anesthesiologists, including one professor holding a PhD, and five nurse anesthetists, with one also being a professor with a PhD. Their expertise ensured that the questionnaire covered relevant themes and maintained alignment with professional standards. Following this expert review, the questionnaire was subjected to pilot testing with board members of the NANA. This group included seven experienced nurse anesthetists, who evaluated the instrument for face and content validity. The testing phase provided practical insights, leading to several critical modifications: (1) Clarification of language: Adjustments were made to improve clarity and eliminate any ambiguous phrasing, ensuring that questions were readily understandable by all respondents, and (2) refinement of question structure: The layout of certain questions was revised to enhance flow and coherence, helping participants navigate the questionnaire with ease and focus.

The final questionnaire consisted of five demographic questions (professional background, additional pediatric education yes/no, being a responsible manager yes/no, workplace activity acute/elective/gynecology/obstetric/pediatric/other, and whether the NSA is implemented in quality improvement/e-handbook/procedures/overall level/education/training of newly employed anesthetic personnel) and 36 statements about the NSA and the 2024 NSA revision. Responses were provided on a Likert scale [9], where 1 = strongly disagree, 2 = disagree, 3 = neither disagree nor agree, 4 = agree, and 5 = strongly agree. Data collection was conducted using a survey solution developed and hosted by the University of Oslo (nettskjema.no).

### 2.4. Analysis

The Statistical Package for the Social Sciences (SPSS) Version 28 was used to analyze the data. Descriptive statistics were used to describe respondents' demographics, with results presented as *n* (%). An independent samples *t*-test was used to compare the responses between anesthetists and nurse anesthetists. The independent samples *t*-test is designed to compare the means of two independent groups, which aligns with many research questions using Likert scale data. Hence, this was deemed suitable for testing whether there were statistically significant differences in mean responses between anesthetists and nurse anesthetists on certain aspects of the NSA. Initially, all datasets were reviewed to identify and categorize missing data. Data gaps were classified as either item nonresponse, where specific questions were left unanswered, or unit nonresponse, where entire responses from certain participants were absent. In instances where missing data were extensive in individual responses, such entries were excluded from the final analysis. No method for imputing missing data was used. A *p* value ≤ 0.05 was considered statistically significant.

### 2.5. Ethical Aspects

The study was based on informed, willing consent to participate. Submission of a completed questionnaire was considered consent to participate. No identifying information was collected, ensuring that individuals cannot be recognized in the presentation of results. Consequently, no specific approvals were required to conduct the study. According to Norwegian legislation, no ethics approval is needed when including healthcare personnel [10].

## 3. Results

In total, 823 respondents completed the questionnaire. This accounts for approximately 14.5 percent of the members of the NAA and 33.6 percent of the members of the NANA.  Table 1 provides an overview of the respondents' characteristics.

As shown in Table 1, the majority of respondents were NANA members (*n* = 605, 73.5%), with only a few having additional pediatric education (*n* = 70, 8.7%) or serving as managers (*n* = 44, 5.4%). Most respondents' workplace practices were acute (*n* = 702, 86.1%), with about half of them involved in gynecological or obstetric activities (*n* = 442, 54.1%), and 43.9% (*n* = 358) having a pediatric ward.  Table 1 also shows that the NSA is primarily implemented in education (*n* = 593, 72.1%), procedures (*n* = 552, 67.1%), and quality improvement (*n* = 491, 59.7%), at an overall level (*n* = 479, 58.2%), and in training of newly employed anesthetic personnel (*n* = 472, 57.4%). Fewer respondents reported implementing the NSA in e-handbooks (*n* = 304, 36.9%).

Table 2 presents the participants' responses to the statements about NSA in the questionnaire.

As shown in Table 2, fewer than 60% of the respondents reported having a good overview of the NSA (*n* = 124, 57.1% to *n* = 356, 59.5%). Anesthesiologists reported a significantly better insight into the 2024 revisions (*n* = 127, 58.3%) than nurse anesthetists (*n* = 275, 46%) (*p*=0.02). In addition, 53.2% of anesthesiologists (*n* = 116) and 55.7% of nurse anesthetists (*n* = 332) reported that the NSA plays a central role in the planning of activities. In total, 19.7% of anesthesiologists (*n* = 43) and 29.1% of nurse anesthetists (*n* = 173) reported being unsure whether the 2024 revision of the NSA had led to changes in clinical practice, with a significant difference between the two groups (*p*=0.02). Moreover, over half of the respondents from both groups reported not noticing any changes due to the NSA revision.

Only 9.7% (*n* = 58, nurse anesthetists) to 12.9% (*n* = 28, anesthesiologists) of respondents, respectively, reported developing local risk and vulnerability analyses for deviations from the NSA, with significant differences between groups (*p*=0.01). Approximately 50% of respondents from both groups reported having a quality system describing training needs and documenting the anesthetic personnel's skills during specialization.

Most respondents indicated that their ward facilitates the maintenance of anesthesia competence (*n* = 440/73.7% of nurse anesthetists to *n* = 168/77.1% of anesthesiologists), and that a consultant anesthesiologist is present continuously (*n* = 463/77.8% of nurse anesthetists to *n* = 143/66.2% of anesthesiologists). In addition, as shown in Table 2, responses indicate areas for improvement to align with the NSA requirements. Fewer than half of respondents reported that their workplace had a focus on environmental considerations, with a significant difference between anesthesiologists and nurse anesthetists (*n* = 70/32.4% of anesthesiologists to *n* = 288/48.4% of nurse anesthetists, *p* < 0.001).

There was a significant difference in responses regarding the importance of developing the NSA in collaboration with the NAA and the NANA (*n* = 175/80.6% of anesthesiologists to *n* = 548/92.1% of nurse anesthetists, *p* < 0.001). Some respondents had been involved in incidents where the NSA was used in evaluations (13.6%–17.1%) or were aware of such incidents (23.2%–30.6%). About two-thirds of the respondents reported having a connection to the NSA in their daily work (65%–69.1%).

## 4. Discussion

The NSA represents the first standard collaboratively developed by anesthesiologists and nurse anesthetists to ensure satisfactory anesthetic practice. This study is the first to report experiences and perspectives on such a standard. Findings demonstrate that while most anesthetic personnel are aware of the NSA and find it relevant to their daily work, there are significant gaps in its perceived impact and implementation. The study highlights areas needing improvement to reach the level of anesthetic practice stipulated by the NSA.

An open question remains whether the NSA truly reflects “satisfactory anesthetic practice” in terms of evidence-based quality and patient safety. Developing a strictly evidence-based guideline was deemed outside the scope due to workload, financing, and lack of evidence. The NSA was designed to address the unique opportunities and challenges in a Norwegian setting, featuring highly trained nurse anesthetists and remote populations [6].

For the 2024 NSA revision, the working group searched the literature, seeking evidence-based guidelines and systematic reviews. However, these searches were not systematic, and the NSA had few references. This is consistent with Rong et al. [11], who noted that clinical practice guidelines in anesthesia often rely on low levels of evidence and have discordant recommendations. Nonetheless, NSA recommendations on preoperative evaluation, monitoring, equipment requirements, and sedation were based on evidence-based ASA practice guidelines [3].

The working group engaged in several discussions, consulting other clinicians, ultimately leading to NSA adoption by the NAA and NANA. Consequently, NSA represents a consensus among these professionals. This consensus should be considered when applying NSA results internationally, where anesthesia care delivery models vary [1].

The NSA has been used in several root cause analyses of adverse anesthetic events in Norway [12]. In our study, about two-thirds of respondents reported engagement with the NSA in daily work, and 13Additionally30 percent had experienced or heard of incidents where the NSA was used in root cause analysis. This suggests NSA's significant impact in Norway.

The study found that 92.1% of nurse anesthetists agreed on the importance of NSA collaboration between the NAA and NANA, compared to 80.6% of anesthetists (*p* < 0.001). This difference might stem from traditional professional jurisdictions and tasks associated with physicians and nurses [13]. Anesthetic work can feature unique tasks or overlapping ones, as noted in the ASA's stance that nurse anesthetists cannot replace physicians, given implications for patient safety [14]. However, studies support nurse anesthetists' contributions to safe and quality anesthetic practice [15, 16].

The NSA delineates task and responsibility division within the anesthetic team. Our experience indicates that collaboration across professional groups is crucial for establishing a consensus-based standard suitable for Norwegian clinical settings. The Norwegian anesthesia model is distinguished by its emphasis on collaboration between anesthesiologists (physicians) and nurse anesthetists, providing a comprehensive framework for task-sharing. This model allows nurse anesthetists to perform a significant portion of anesthetic tasks, under the oversight of anesthesiologists. Globally, anesthesia practices vary widely, influenced by factors such as healthcare systems, regulatory environments, and available resources [1]. In many countries, anesthesiologists primarily handle anesthesia tasks, with limited involvement from nurse anesthetists [2]. In contrast, the Norwegian model reflects a more integrated approach, enabling nurse anesthetists to play a vital role in delivering anesthesia services. The task-sharing framework ensures that trained nurse anesthetists can perform critical procedures, thereby maintaining the continuity and quality of care. This collaborative practice model could serve as inspiration for other countries with similar geographic and healthcare delivery challenges. However, its success also hinges on factors such as extensive training for nurse anesthetists and a robust support system, which may not be feasible in all countries.

We propose that our national standard could be locally adapted and adopted internationally given the overlapping roles of anesthesiologists and nurse anesthetists. Developing similar standards in settings with overlapping professional jurisdictions may be beneficial. However, the applicability of the results and the NSA to international contexts should consider varying regulatory environments and training protocols for anesthesia personnel.

### 4.1. Limitations

One significant limitation of this study is the low and unequal response rates, which may introduce response bias. With only a fraction of the targeted anesthetic personnel participating, the results might not accurately represent the broader perspectives of all professionals impacted by the NSA. This uneven participation can skew the findings, as the views of those who chose to respond may differ from those who did not. The low response rate limits the generalizability of the results, raising uncertainty about whether the respondents' views reflect those of the entire population of anesthesiologists and nurse anesthetists. Moreover, the response rates varied between the two groups, with 14.5% of members from the NAA responding, compared to 33.6% from the NANA. Despite these differences, the similarity in responses between anesthesiologists and nurse anesthetists and the relatively large sample size suggest that the findings are transferable across Norwegian clinical settings.

In addition, self-selection bias presents another critical limitation. Individuals with a positive inclination toward the NSA or those who perceive it as valuable might be overrepresented in the responses. Consequently, the survey may disproportionately capture favorable opinions, overlooking potential criticisms or concerns held by those who opted not to participate. This bias can result in an overly optimistic portrayal of the NSA's acceptance and effectiveness, impacting the study's credibility and limiting its usefulness in identifying areas needing improvement.

Another limitation is the lack of a validated tool to assess experiences and perspectives. However, the questionnaire was developed and reviewed by experts, defined as acknowledged in the field of anesthesia, and piloted to ensure face and content validity.

The quantitative design entails the limitation of not providing in-depth information-only responses to the predefined questions. There is also a potential self-selection bias, and respondents with a prior interest in the NSA may be overrepresented.

There is ongoing debate regarding the application of parametric tests, such as the *t*-test, to ordinal data, like those generated from Likert scales. Critics argue that Likert scale responses, being ordinal, do not meet the assumptions required for parametric testing, such as the interval level of measurement. However, some researchers advocate for the use of parametric tests when the data meet other criteria, such as a sufficiently large sample size and adequate approximation to a normal distribution, which can justify the treatment of ordinal data as interval-level data. In this study, the sample size and distribution characteristics were considered appropriate for the application of the *t*-test. Nonetheless, researchers exercised caution in interpreting results, understanding that such statistical methods provide insights into possible trends or differences between groups but may not capture the nuances inherent to ordinal data.

## 5. Conclusion

This study is pioneering in exploring the experiences and perspectives of anesthesiologists and nurse anesthetists concerning a national standard for safe anesthesia practice. While the NSA is context-sensitive and relevant to the Norwegian setting, our findings reveal areas that require improvement to align fully with satisfactory anesthetic practice. Notably, the NSA's limited evidence base may impact its credibility and acceptance, raising questions about its effectiveness in driving consistent practice standards.

### 5.1. Implications

To deepen the understanding of the NSA's effectiveness and application, future research endeavors should incorporate qualitative interviews targeting both anesthetic personnel and healthcare managers. These interviews should focus on identifying specific barriers to NSA adoption, clarifying roles within the anesthesia team, and assessing training needs that align with the standard. Such qualitative insights will reveal practical challenges and facilitate the development of targeted strategies to enhance NSA implementation.

Furthermore, the collaboration between the NAA and NANA in creating an assessment tool presents a promising approach to ensuring compliance with NSA requirements. This tool should incorporate features such as a checklist for self-evaluation, criteria for best practices, and rubrics for conducting risk and vulnerability analyses at local levels. These elements will empower managers and professionals to proactively refine their anesthetic practices in accordance with NSA standards.

In addition, the findings from this study should inform the ongoing training and educational programs for anesthesiologists and nurse anesthetists. Targeted curricula should address the areas identified as misaligned with NSA standards, promoting a culture of continuous improvement and adaptation.

While the NSA offers a useful framework for enhancing anesthetic practice within Norway, it may hold international relevance as healthcare systems seek effective models of interprofessional collaboration. However, its applicability should be considered alongside variations in regulations and healthcare structures that exist globally. Recognizing these differences is crucial to adapting the NSA to suit diverse contexts, ensuring it effectively supports safe and high-quality anesthetic care worldwide.

## Figures and Tables

**Table 1:** Respondent's characteristics (*N* = 823).

	*n* (%)
*Professional background*	
Consultant anesthesiologist	172 (20.9)
Registrar anesthesiologist	46 (5.6)
Nurse anesthetist	560 (68)
Student nurse anesthetist	22 (2.7)
Retired nurse anesthetist	14 (1.7)
Nurse anesthetist academic	9 (1.1)

*Additional pediatric education*	
SSAI	15 (1.9)
NUBA	24 (3)
Other	31 (3.8)

*Manager*	
Yes	44 (5.4)

*Hospital activity*	
Acute	702 (86.1)
Elective	243 (29.8)
Gynecology/obstetrics	442 (54.2)
Pediatric ward	358 (43.9)
Education	442 (54.2)
Other	61 (7.5)

*NSA implemented in:*	
Quality improvement	491 (59.7)
E-handbook	304 (36.9)
Procedures	552 (67.1)
Overall level	479 (58.2)
Education	593 (72.1)
Training newly employed	472 (57.4)

*Note:* SSAI = Scandinavian Society of Anesthesiology and Intensive Care Medicine. NUBA = Nordic Education in Pediatric Anesthesia for Nurse Anesthetists. NSA = Norwegian Standard for the Safe Practice of Anesthesia.

**Table 2:** Anesthesiologists' and nurse anesthetists' responses to the questionnaire statements (*N* = 816).

	Nurse anesthetist (*n* = 598)				Anesthetist (*n* = 218)				*p* value
	Disagree *n* (%)	Neither/nor *n* (%)	Agree *n* (%)	Unsure *n* (%)	Disagree *n* (%)	Neither/nor *n* (%)	Agree *n* (%)	Unsure *n* (%)	
I have good overview of the NSA	89 (14.9)	140 (23.4)	356 (59.5)	13 (2.1)	37 (17.1)	53 (24.4)	124 (57.1)	3 (1.4) *M* = 1	0.32
I have good overview of the NSA 2024 revisions	139 (23.2)	160 (26.8)	275 (46)	24 (4)	33 (15.1)	54 (24.8)	127 (58.3)	4 (1.8)	0.02^∗^
I only have overview of chapters of the NSA of specific relevance to my work	176 (29.5)	144 (24.1)	257 (43)	20 (3.4) *M* = 1	73 (33.5)	44 (20.2)	96 (44)	5 (2.3)	0.48
In my ward, NSA is not used until adverse events occur	308 (51.7)	126 (21.1)	44 (7.4)	118 (19.8) *M* = 2	112 (51.9)	42 (19.4)	27 (12.5)	35 (16.2)	0.81
In my ward, NSA is leading the planning of our activity	54 (9.1)	111 (18.6)	332 (55.7)	99 (16.6) *M* = 2	27 (12.4)	47 (21.6)	116 (53.2)	28 (12.8)	0.40
In my ward, NSA 2024 has led to changes in our practice	169 (28.5)	143 (24.1)	109 (18.4)	173 (29.1) *M* = 4	76 (34.9)	50 (22.9)	49 (22.5)	43 (19.7)	0.02^∗^
I have not noticed any changes due to the 2024 NSA revision	96 (16.1)	119 (20)	299 (50.2)	82 (13.8) *M* = 2	48 (22)	35 (16.1)	116 (53.2)	19 (8.7)	0.08

*In my ward, we work in-line with the NSA regarding…*									
Having developed local risk and vulnerability analyses for deviations from the NSA	113 (19)	110 (18.5)	58 (9.7)	314 (52.8) *M* = 3	61 (28.1)	33 (15.2)	28 (12.9)	95 (43.8)	0.01^∗^
Having a quality system describing training needs, and documentation of anesthetic personnels' skills at different levels of specialization	80 (13.5)	70 (11.8)	316 (53.4)	126 (21.3) *M* = 6	42 (19.4)	31 (14.3)	113 (52.1)	31 (14.3)	< 0.01^∗^
Facilitating maintenance of anesthesia competence (e.g., team training and simulation)	63 (10.6)	65 (10.9)	440 (73.7)	29 (4.9) *M* = 1	23 (10.6)	25 (11.5)	168 (77.1)	2 (0.9)	0.42
Having a consultant anesthesiologists present continuously	103 (17.3)	14 (2.4)	463 (77.8)	15 (2.5) *M* = 3	62 (28.7)	9 (4.2)	143 (66.2)	2 (0.9) *M* = 2	< 0.001^∗^
Inspection and use of medical equipment	18 (3)	52 (8.7)	395 (66.4)	130 (21.8) *M* = 3	7 (3.2)	16 (7.3)	132 (60.6)	63 (28.9)	0.41
Preoperative evaluation and information	16 (2.7)	54 (9)	425 (71.2)	102 (17.1) *M* = 1	7 (3.2)	25 (11.5)	149 (68.7)	36 (16.6) *M* = 1	0.41
Having implemented the frailty score for persons ≥ 65 years of age	155 (26.1)	101 (17)	191 (32.2)	147 (24.7) *M* = 4	76 (35.2)	48 (22.2)	72 (33.3)	20 (9.3) *M* = 2	< 0.001^∗^
Monitoring and equipment requirements in anesthesia	21 (3.5)	42 (7.1)	482 (81)	50 (8.4) *M* = 3	9 (4.2)	13 (6)	166 (76.9)	28 (13) *M* = 2	0.33
Having a routine to use capnography in nonconscious sedation	166 (27.9)	107 (18)	297 (49.8)	26 (4.4) *M* = 2	33 (15.1)	52 (23.9)	124 (56.9)	9 (4.1)	< 0.001^∗^
Having a routine to use neuromuscular monitoring when using nondepolarizing muscle relaxants	29 (4.9)	37 (6.2)	517 (86.6)	14 (2.3) *M* = 1	12 (5.6)	13 (6)	188 (87)	3 (1.4) *M* = 2	0.60
Focusing on environmental considerations	101 (17)	176 (29.6)	288 (48.4)	30 (5) *M* = 3	62 (28.7)	74 (34.3)	70 (32.4)	10 (4.6) *M* = 2	< 0.001^∗^
Staffing	68 (11.4)	107 (18)	258 (43.4)	162 (27.2) *M* = 3	34 (15.6)	41 (18.8)	98 (45)	45 (20.6)	0.04^∗^
Pediatric anesthesia	33 (5.5)	66 (11.1)	399 (67.1)	97 (16.3) *M* = 3	25 (11.5)	30 (13.8)	131 (60.4)	31 (14.3) *M* = 1	< 0.01^∗^
Anesthesia outside operating theater departments	22 (3.7)	97 (16.4)	313 (52.9)	160 (27) *M* = 6	9 (4.2)	28 (13)	130 (60.2)	49 (22.7) *M* = 2	0.76
Sedation	18 (3)	86 (14.4)	361 (60.6)	131 (22) *M* = 2	10 (4.6)	34 (15.7)	124 (57.4)	48 (22.2) *M* = 2	0.45
Obstetric anesthesia^∗∗^	13 (2.2)	84 (14.3)	326 (55.5)	164 (27.9) *M* = 11	6 (2.8)	22 (10.2)	136 (63.3)	51 (23.7) *M* = 3	0.82
Anesthetic work outside the hospital	13 (2.2)	99 (16.7)	129 (21.8)	351 (59.3) *M* = 6	2 (0.9)	38 (17.5)	55 (25.3)	122 (56.2) *M* = 1	0.84
Anesthetic work in intrahospital emergencies and intensive care units	6 (1)	86 (14.5)	247 (41.7)	253 (42.7) *M* = 6	6 (2.8)	27 (12.4)	121 (55.8)	63 (29) *M* = 1	0.01^∗^
Documentation	21 (3.5)	54 (9.1)	412 (69.2)	108 (18.2) *M* = 3	7 (3.2)	22 (10.1)	135 (61.9)	54 (24.8)	0.23
Reporting of anesthesia-related problems and complications	15 (2.5)	72 (12.1)	369 (62.1)	138 (23.2) *M* = 4	5 (2.3)	15 (6.9)	138 (63.3)	60 (27.5)	0.06
Monitoring after anesthesia	9 (1.5)	40 (6.7)	447 (75.1)	99 (16.6) *M* = 3	4 (1.9)	10 (4.6)	172 (79.6)	30 (13.9) *M* = 2	0.75
Requirements for same day discharge	5 (0.8)	57 (9.6)	237 (39.8)	296 (49.7) *M* = 3	5 (2.3)	13 (6)	125 (57.3)	75 (34.4)	< 0.01^∗^
I received good information in relation to the hearing of the 2024 NSA revision	119 (20)	155 (26)	267 (44.8)	55 (9.2) *M* = 2	35 (16.1)	56 (25.7)	114 (52.3)	13 (6)	0.49
I provided input to the hearing of the 2024 NSA revision	330 (55.6)	141 (23.7)	92 (15.5)	31 (5.2) *M* = 4	115 (53)	35 (16.1)	58 (26.7)	9 (4.1) *M* = 1	0.11
It is important that the NSA is developed in collaboration between the NAA and the NANA	3 (0.5)	19 (3.2)	548 (92.1)	25 (4.2) *M* = 3	6 (2.8)	29 (13.4)	175 (80.6)	7 (3.2) *M* = 1	< 0.001^∗^
I have been involved in an incident where NSA has been used in the evaluation	345 (58.1)	90 (15.2)	81 (13.6)	78 (13.1) *M* = 4	133 (61.3)	23 (10.6)	37 (17.1)	24 (11.1) *M* = 1	0.65
I have heard about incidents where NSA has been used in the evaluation	209 (35.2)	95 (16)	138 (23.2)	152 (25.6) *M* = 4	81 (37.5)	27 (12.5)	66 (30.6)	42 (19.4) *M* = 2	0.44
I have no relation to the NSA in my daily work	412 (69.1)	107 (18)	63 (10.6)	14 (2.3) *M* = 2	141 (65)	40 (18.4)	34 (15.7)	2 (0.9)	0.44

*Note:* NSA, Norwegian Standard for the Safe Practice of Anesthesia; NAA, Norwegian Association of Anesthesiologists; NANA, Norwegian Association of Nurse Anesthetists. Independent samples *t*-test. M = missing. Response alternatives strongly disagree/disagree = disagree, agree/strongly agree = agree.

^∗^ *p* < 0.05 = statistically significant.

^∗∗^Only 442 hospitals had gynecological/obstetric wards.

## Data Availability

Data are available upon reasonable request to the first author.

## Supporting Information

STROBE statement, a checklist of items that should be included in reports of observational studies.
